# Contents of the Present Number

**Published:** 1867-10

**Authors:** 


Contents of the Present Number.—We furnish our readers this month with the most "*original*" article we have yet published, and hope they will observe the "evolution" of ten new commandments. Our friend, Dr. C., has figured matrimonial "amours" down to a very fine point. but he quotes authority which is unquestioned and his conclusions are logical. The idea that Job's mother knew the night he was conceived, "that a male child was conceived," reminds us of the famous charlatan who based diagnosis altogether upon the appearance of the urine. and was one day presented with a specimen and requested to be very minute and careful in his examination, as it was a very critical and obscure case of disease. After considerable delay and great show of careful observation he announced, "de patient is two or three days pregnant, and de fœtus has de worms."

